# To what extent does income explain the effect of unemployment on mental health? Mediation analysis in the UK Household Longitudinal Study

**DOI:** 10.1017/S0033291722003580

**Published:** 2022-12-01

**Authors:** Rachel M. Thomson, Daniel Kopasker, Alastair Leyland, Anna Pearce, S. Vittal Katikireddi

**Affiliations:** MRC/CSO Social and Public Health Sciences Unit, University of Glasgow, Glasgow, UK

**Keywords:** anxiety, depression, employment, health inequalities, income, public health

## Abstract

**Background:**

Employment and income are important determinants of mental health (MH), but the extent that unemployment effects are mediated by reduced income is unclear. We estimated the total effect (TE) of unemployment on MH and the controlled direct effect (CDE) not acting via income.

**Methods:**

We included adults 25–64 years from nine waves of the UK Household Longitudinal Study (*n* = 45 *497*/*obs* = 202 297). Unemployment was defined as not being in paid employment; common mental disorder (CMD) was defined as General Health Questionnaire-12 score ≥4. We conducted causal mediation analysis using double-robust marginal structural modelling, estimating odds ratios (OR) and absolute differences for effects of unemployment on CMD in the same year, before (TE) and after (CDE) blocking the income pathway. We calculated percentage mediation by income, with bootstrapped standard errors.

**Results:**

The TE of unemployment on CMD risk was OR 1.66 (95% CI 1.57–1.76), with 7.09% (6.21–7.97) absolute difference in prevalence; equivalent CDEs were OR 1.55 (1.46–1.66) and 6.08% (5.13–7.03). Income mediated 14.22% (8.04–20.40) of the TE. Percentage mediation was higher for job losses [15.10% (6.81–23.39)] than gains [8.77% (0.36–17.19)]; it was lowest for those 25–40 years [7.99% (−2.57 to 18.51)] and in poverty [2.63% (−2.22 to 7.49)].

**Conclusions:**

A high proportion of the short-term effect of unemployment on MH is not explained by income, particularly for younger people and those in poverty. Population attributable fractions suggested 16.49% of CMD burden was due to unemployment, with 13.90% directly attributable to job loss rather than resultant income changes. Similar analytical approaches could explore how this differs across contexts, by other factors, and consider longer-term effects.

## Introduction

Employment influences mental health (MH) both for those with existing psychiatric disorders and for the general population (Evans & Repper, 2000). There is a well-documented association between job loss and increased likelihood of common MH problems such as depression (Paul & Moser, 2009). Moving into employment also has a protective effect on MH (Modini et al., 2016), more so than exists for general health or mortality (Gathergood, 2013).

The financial strain theory of unemployment suggests that a critical factor in understanding this relationship is the degree to which changes in employment influence an individual’s health via altered income (Ervasti & Venetoklis, 2010; Kessler, Turner, & House, 1988). This is highly plausible: income is inversely related to poor MH in the population (Marmot, 2005), and individual-level changes in income have been shown to be associated with future MH and wellbeing (Thomson et al., 2022). However, given that it is improbable one’s employment status could change *without* a subsequent change in income, untangling the effects of these exposures from one another is methodologically challenging. There is additional complexity added by the reciprocal relationship between MH and employment: poor MH is likely both a consequence and cause of unemployment (Olesen, Butterworth, Leach, Kelaher, & Pirkis, 2013).

To overcome these issues, use of causally-informed methods to investigate mediation and more accurately account for time-varying confounding can be helpful (VanderWeele, 2009). These allow us to consider and quantify how the *total effect* (TE) of unemployment on MH (which would include any effect exerted via income changes) may differ from the *direct effect* (which blocks the causal pathway via income). Such information is clearly of value to policymakers: the former gives an indication of the potential effects of employment policies on population MH, whereas the latter indicates to what extent income support policies might alleviate the MH impacts of unemployment. We therefore aimed to estimate and compare the total and direct effect of unemployment on MH, using data representative of the UK working-age population. We hypothesised that income would explain a relatively high proportion of the unemployment effect, and that the extent of this mediation would be likely to differ between population subgroups.

## Methods

### Participants

Data were from the UK Household Longitudinal Study (UKHLS), a nationally representative sample of around 40 000 households followed annually since 2009 (University of Essex, Institute for Social & Economic Research, NatCen Social Research, & Kantar Public, 2020). We included all working-age adults aged 25–64 years (excluding those <25 to exclude the majority of those in ongoing education and to avoid misclassifying highest educational attainment) and used nine waves of data up to 2019 (to reduce potential confounding effects of the COVID-19 pandemic).

### Variables

#### Exposure and outcome

Our exposure of interest was a binary measure of whether an individual was (employed) or was not (unemployed) in any form of paid employment at each wave of data collection. Given our focus on mediation by income, we selected this measure focusing specifically on experiences of paid work to ensure we included all those in our working-age sample whose incomes would be directly impacted by a change in employment status. Prevalence of poor MH was assessed in each wave using the General Health Questionnaire-12 (GHQ-12), a validated screening tool for symptoms of psychological distress used widely in epidemiological research (Goldberg et al., 1997). It generates a score between 0 and 12, with four or above indicating a strong likelihood of common mental disorder (CMD), which we refer to subsequently as ‘caseness’.

#### Mediator and confounders

We measured both continuous income and whether an individual lived in relative poverty, as our previous work has demonstrated these are likely to exert distinct effects (Kromydas, Thomson, Pulford, Green, & Katikireddi, 2021). We generated a binary variable indicating whether individuals were living in a household which was above or below the poverty line at each timepoint, defined as household equivalised income <60% median after housing costs. To account for non-normality and the fact that the income-health relationship is thought to be curvilinear (Mackenbach et al., 2004), continuous income was operationalised in analyses as the log of OECD equivalised household income.

**Fig. 1:**
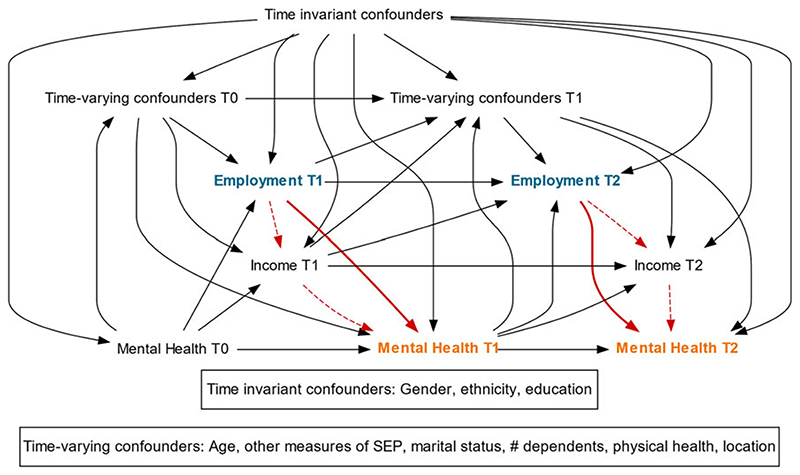
Causal diagram of the hypothesised causal relationships between employment and MH, mediated by income. Exposure = employment (blue), mediator = income, and outcome = MH (orange). Dashed red line illustrates indirect/mediated pathway to outcome via income; bold red line illustrates direct effect. SEP = socioeconomic position.

To explicitly illustrate the hypothesised causal pathway between our exposure and outcome, we generated a directed acyclic graph (DAG) based on existing literature which informed our statistical analysis (Fig. 1). We examined the association between unemployment and CMD within each sweep (after adjusting for prior CMD), therefore estimating the short-term impacts of unemployment on CMD. We identified the minimally sufficient adjustment set for the short-term effect of employment on MH at a timepoint (t) as being: all time invariant confounders; timevarying confounders at *t*−1; income at *t*−1; employment at *t*−1; and MH at *t*−1.

We measured our identified time-invariant or baseline confounders as self-reported gender (male/female), ethnicity (White/non-White), and highest educational attainment [high (degree-level), medium (GCSE, A-level or equivalent) or low (no formal qualifications)]. Time-varying confounders were housing tenure (owner/renter); receipt of welfare benefits (yes/no); relationship status (single/coupled); number of children <16 in the household; physical health [continuous score from the shortform survey (SF-12) physical health component (PCS)] (Gandek et al., 1998); and location (government office region). Age was also considered a time-varying covariate, since the spacing between sweeps varied from household to household, and was included as a continuous variable and a squared term. As can be seen from the DAG, the same intermediate confounders were identified for employment and income, because we considered the impact of employment on income to be instantaneous. Therefore, there were no identified exposure-induced mediator outcome confounders.

Based on the DAG, time-varying confounders were included in all modelling as one-year lagged terms, to ensure that they preceded the exposure. To ensure analyses adequately corrected for the influence of past exposure and past outcome status (Fig. 1), one-year lagged versions of employment status, GHQ caseness, and the MH component of SF-12 were also included as timevarying confounders.

### Statistical analysis

#### Primary analysis

We used a double-robust marginal structural modelling approach, which incorporates the relationships expressed in the DAG to generate the desired causal estimates, under the assumption of no residual/unmeasured confounding not captured by the DAG or able to be included from available data (Robins, Hernán, & Brumback, 2000; VanderWeele, 2009). Using a double-robust estimator ensures that results are unbiased if either the exposure or outcome model is correctly specified.

We first calculated stabilised inverse probability of treatment weights (IPTWs) in an exposure model with all identified time invariant and lagged time-varying confounders, which aims to create exchangeability between exposed and unexposed individuals (as would exist following randomisation in a sufficiently large trial). We tested IPTW performance by comparing the standardised mean differences (SMDs) of confounding variables between groups pre- and post-weighting; SMD <0.1 was judged to indicate a negligible statistical difference and SMD <0.2 reasonable balance (Nguyen et al., 2017). We then applied these weights in an outcome model using pooled logistic regression, including adjustment for baseline and time-varying confounders. We calculated odds ratios (OR) and absolute risk differences in percentage points. We also calculated population attributable fractions (PAFs) to determine the percentage of CMD burden attributable to unemployment according to our causal estimate (Mansournia & Altman, 2018):

$$\begin{array}{lll} PAF\; & = & \frac{\;Observed\;cases\;-\;Expected\;cases\;}{\;Observed\;cases\;}\\ & = & \frac{\;Prevalence\;in\;total\;population\;-\;Prevalence\;in\;unexposed\;}{\;Prevalence\;in\;total\;population\;} \end{array}$$

The TE of unemployment was calculated by including only lagged measures of income within the IPTW weighting and regression modelling (Fig. 1). When calculating the *controlled direct effect* (CDE) of unemployment, concurrent measures of income were additionally added in the weight to block the pathway between unemployment and MH operating via income change. The degree of mediation by income is expressed as the percentage attenuation of the TE once the pathway via income is blocked: ‘100 × (TE – CDE)/TE’. We calculated 95% confidence intervals around this percentage using bootstrapping with 1000 re-samplings (Stringhini et al., 2012).

#### Sensitivity and subgroup analyses

As it has been hypothesised that job losses may have a larger magnitude of effect for MH than job gains (Paul & Moser, 2009), we conducted an additional analysis where the exposure variable was a transition into/out of unemployment since the previous wave of data collection, restricting the sample to only those at risk of this exposure. We also investigated the role of four potential effect modifiers using stratified analyses: whether an individual lived in poverty; gender; educational attainment; and age dichotomised into younger (25–40 years) and older (41–64 years) working-age. Finally, we performed a complete case analysis for comparison with our imputed sample. As an additional sensitivity analysis, in this complete case sample we also re-ran our analysis using the more traditional International Labour Organisation (ILO) definition of unemployment (separating those who are ‘economically inactive’, such as homemakers or early retirees, from those who are unemployed) to ensure our selection of exposure measurement focused solely on whether someone did/did not report being in paid work did not unduly influence our results.

#### Multiple imputations

We used multiple imputations with chained equations to address any missing data between an individual’s first and last appearance in UKHLS under a missing at random assumption (Desai, Esserman, Gammon, & Terry, 2011; White, Royston, & Wood, 2011). Observations with high missingness (those missing >9 of the 22 variables required for analysis, totalling 12.7% of all observations) were dropped. Due to the need for lagged data on time-varying confounders, the first wave of data for each individual only contributed information about baseline confounding characteristics. Twenty imputed datasets were then created with all variables including exposure, mediator, outcome, and lagged time-varying confounders.

Gender, age, wave, and number of children were used as imputation variables; due to issues achieving model convergence missing observations for region (n = 211, 0.0007%) were dropped. Poverty and caseness variables were dichotomised after imputation. Online Table S1 in the supplementary appendix details the regression models used to impute each included variable.

Stata MP 16.1 was used for all analyses. Graphs were generated in R using ggplot2.

## Results

The analytic sample included 45 497 individuals across 202 297 observations; of these, 70.6% (n = 32 138; obs = 132 962) had complete data (see Fig. S1 in appendix for detail on participant exclusion). Mean age was 45.31 (S.D. 10.93), 55.3% of observations were from women, and 25.7% of observations were from those not in paid work in that wave (online Supplementary Table S2, appendix). The mean income loss among those who experienced new unemployment was £295.20/month (18.9% of the average monthly household income in the sample), with mean increase following job gain £304.07/month. Compared with those who gained jobs (*n* = 7399), those who lost jobs during the study period (*n* = 7984) were slightly older (mean age 47.4 *v*. 42.8), were less likely to be in poverty in the preceding year (22.4% *v*. 45.7%), and were more likely to be homeowners (69.8% *v*. 59.0% – see online Supplementary Table S3 for all characteristics of those exposed). The imputed sample included slightly more men, non-White individuals, and those from lower socioeconomic backgrounds, and had higher prevalence of the exposure, mediator, and outcome.

### Balance of confounding variables

**Fig. 2:**
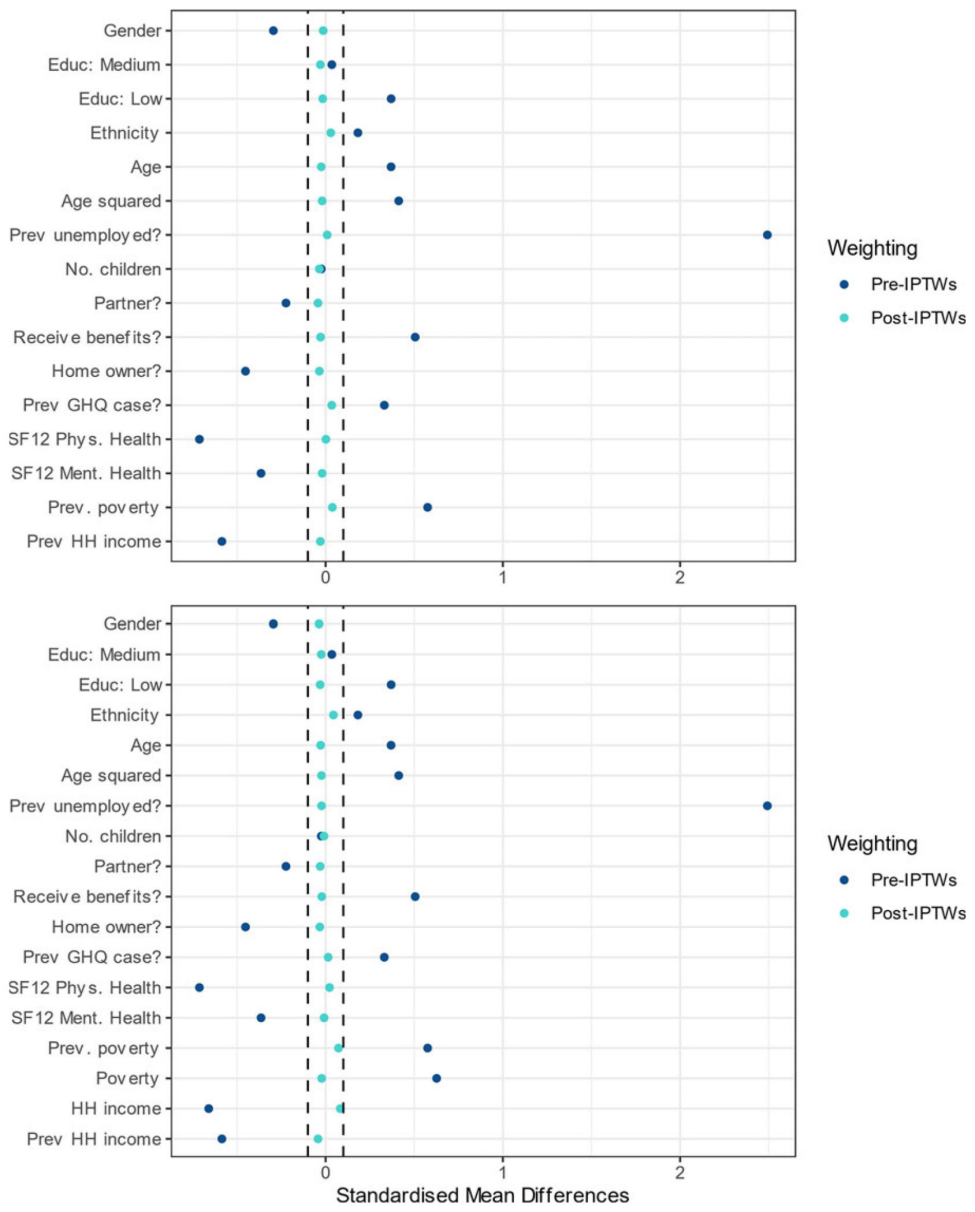
Balance of confounding variables before and after applying inverse probability of treatment weights (IPTWs) in primary analysis for total effect (top panel) and controlled direct effect (bottom panel).

Good exchangeability was achieved in the primary analysis investigating the total and direct effect of the exposure with all SMDs <0.1 after application of IPTWs, indicating statistically negligible difference (Fig. 2 and online Supplementary Tables S4/S5 in appendix; region only illustrated in tables for brevity). Prior to application of weights, previous employment status was the most unbalanced characteristic between exposed and unexposed groups (SMD 2.49). In the stratified sensitivity analyses, for the TE all post-weighting SMDs were also <0.1, though for the direct effect some SMDs for income or poverty variables still remained slightly above this after weighting (maximum 0.14, see online Supplementary Table S5).

### Primary analysis

Expressed in relative terms, the TE of unemployment on likelihood of CMD is OR 1.66 (95% CI 1.57–1.76). This effect size is attenuated when the path via concurrent income is blocked, with the direct effect of unemployment measuring OR 1.55 (1.46–1.66) (Table 1). A similar relationship is seen using absolute measures, with those exposed to unemployment having a prevalence of CMD 7.09% higher than those who are employed when measuring the TE (95% CI 6.21–7.97) *v*. 6.08% (5.13–7.03) for the direct effect. The percentage mediation, indicating the proportion of the TE explained by the mediator, was 14.22% (8.04–20.40). The PAFs indicate that, if our findings represent true causal estimates, 16.49% of the burden of CMD in the UK working-age population during the study period was due to unemployment, with most of the CMD burden (13.90%) directly attributable to unemployment rather than income changes resulting from job loss.

### Sensitivity analysis

When separating moves into employment from moves out of employment, only small differences were seen in effect magnitude: the TE of a job gain was −6.35% decrease in likelihood of CMD (95% CI −7.83 to −4.86) compared with an increased likelihood of 7.42% (6.38–8.47) for a job loss (Table 2). Percentage mediation of the effect by income was higher for job losses than job gains, though confidence intervals were wide: 15.10% (6.81–23.39) *v*. 8.77% (0.36–17.19). The PAFs indicate the direct effect of job gain accounted for a 11.19% reduction in prevalence of CMD among those who were previously unemployed, and direct effect of job losses resulted in a 15.90% increase in prevalence in CMD among those employed in the previous year.

Considering potential effect modifiers, when stratifying by gender effects were larger for men than women: for the direct effect of unemployment, percentage difference 7.83% (6.19–9.47) *v*. 4.98% (3.81–6.15) (Table 3). There was a gradient in effect size by education, with the direct effect ranging from 5.22% (3.65–6.80) for those with the highest education to 7.41% (5.30–9.53) for the least educated. Stratifying by age, effects were larger in younger than older working-age adults, particularly for the direct effect [percentage difference 7.10% (5.39–8.82) *v*. 5.71% (4.50–6.91)]. Effects were also higher for those who were in poverty in the same sweep as employment was measured: for the direct effect, percentage change 8.61% (7.09–10.14) *v*. 5.15% (3.97–6.33) for those who were not in poverty.

The percentage of the unemployment effect mediated by income was greatest for those who were of older working-age [17.97% (9.95–25.99)] and markedly lower for those of younger working-age [7.99% (−2.57 to 18.55)]. There was a slight gradient in percentage mediation by education [from 13.60% (−0.13 to 27.33) in the most educated to 15.25% (2.52–27.97) in the least educated] but confidence intervals were very wide. The degree of mediation for those in poverty after the employment change was particularly small compared with all other groups: 2.63% (-2.22 to 7.49).

Results from the complete case analysis were similar to those estimated from the imputed sample (online Supplementary Table S6), though percentage mediation was slightly higher at 17.73% (9.12–26.34), and balance of confounders was less successful for the direct effect, implying the complete cases could not be balanced as well on the mediator (online Supplementary Table S5). Our sensitivity analysis excluding those who did not meet the ILO definition of employed or unemployed (online Supplementary Table S7) found that, while both the total and direct effect sizes for unemployment were considerably higher using this measure (OR 2.60 for the TE, *v*. 1.66 in the primary analysis), percentage mediation by income was very similar if less precise [13.45% (3.37–23.53) *v*. 14.22% (8.04–20.40) in the primary analysis], suggesting that our use of a less commonly used measure of employment status focused only on experiences of paid work did not unduly influence results for our primary outcome.

## Discussion

In our representative UK-based sample, unemployment resulted in a 7.1% short-term increase in prevalence of common MH problems (OR 1.66). When the pathway via income was blocked this reduced to 6.1% (OR 1.55), suggesting that 14.2% of the effect of unemployment on MH was mediated via income (though the estimate was imprecise). Our findings suggest that the direct effect of unemployment alone explains 13.9% of the burden of poor MH in the UK population, with an additional 2.6% explained by the mediated pathway via income. There was no clear difference in effect magnitude for job losses compared with job gains, but income mediated more of the effect for job losses (15.1% *v*. 8.8%). The degree of mediation also varied between population groups, with income explaining from 2.6% to 18.0% of the TE of unemployment. Income may be a more important part of the causal pathway for older people and potentially for the lowest educated, while those of younger working-age and those in poverty seem to experience a relatively larger direct effect of unemployment on their MH. The TE of employment was largest for men, for the least educated, for those of younger working-age, and for those in poverty after the employment change.

Our findings on the TE size of unemployment are broadly in keeping with existing literature, where the effects of unemployment on MH have been subject to much study for many years. (Warr, Jackson, & Banks, 1988) Paul and Moser’s seminal meta-analysis of employment and MH found that job loss was associated with an OR of 1.57 (*d* = 0.25) for worsened MH after excluding cross-sectional studies (Paul & Moser, 2009); Van der Noort in a similar meta-analysis of prospective studies found that job gain was associated with an OR of 0.52 for depression and 0.79 for psychological distress (van der Noordt, H, Droomers, & Proper, 2014). However, there is less pre-existing consensus in the literature regarding the extent to which income mediates the relationship between unemployment and poorer health or wellbeing, with much work now being several decades old (Kessler et al., 1988; Whelan, 1992).

Using European data, Ervasti and Venetoklis investigated two key theoretical models: the deprivation theory (where the effect of job loss is a key psychological stressor independent of its financial effects) and the financial strain theory (where income loss is the most important aspect of the unemployment effect) (Ervasti & Venetoklis, 2010). They concluded that while both theories have merit, in most countries financial strain was the most important factor driving differences in wellbeing between those who were employed and unemployed, though their use of cross-sectional data precluded causal conclusions. In contrast, and more in keeping with our own findings, Tøge in more robust fixed-effects analyses of European panel data found that the effect of employment changes on self-rated health [which incorporates aspects of both mental and physical health (Singh-Manoux et al., 2006)] attenuated by only 19% after accounting for subjective financial strain; she found no clear evidence for a mediating effect for more objective income measures (Tøge, 2016).

Our findings are less aligned with the only other work we are aware of investigating this question using similar statistical methods to our own by Bijlsma et al. (Bijlsma, Tarkiainen, Myrskylä, & Martikainen, 2017). Applying g-formula calculation to Finnish register data, despite reporting a similar-sized total unemployment effect on first antidepressant prescription (–7.6%) they found that 59% of this effect was mediated by income, and that the CDE of employment was small and non-significant with a hazard ratio of 0.97. It is possible that this may represent a true difference in the extent to which income mediates the employment effect in a UK context [where unemployment benefits are among the lowest of all OECD countries (OECD, 2022)] compared with the more comprehensive welfare support system in the Finnish context. This would indicate that, paradoxically, the direct effect of unemployment on MH may be larger in countries where the experience of unemployment is typically accompanied by significant financial loss, perhaps through pathways relating to stigma and loss of social status (Ervasti & Venetoklis, 2010). However, it is difficult to draw definitive conclusions based on simple comparison of these two studies, and further work would be of interest. Neither Tøge nor Bijlsma differentiated between job losses and job gains (Bijlsma et al., 2017; Tøge, 2016), so it would also be interesting to see whether our finding of greater income mediation for losses is repeated in other contexts.

Considering our other proposed effect modifiers, there is debate in the literature around how socioeconomic position (SEP) may influence sensitivity to episodes of unemployment. Turner argued that, for those of lower SEP, the key mechanism of the relationship between unemployment and MH is likely to be financial strain, whereas for those of higher SEP the mechanism is more likely to operate via threats to self-concept and status (Turner, 1995). Our findings lend some credence to this: while we report larger TEs for those with least education, the degree of mediation by income was somewhat smaller for those in the most educated group (13.6% compared with 15.3%), though wide confidence intervals around these estimates preclude strong inferences. Our most convincing stratified finding is perhaps the difference between those of younger and older working-age, where only 8.0% of the unemployment effect is mediated by income for those aged 25–40, compared with 18.0% for those aged 41–64. We are not aware of other studies having considered age as a potential effect modifier of this relationship, and it would be useful to explore this in other research contexts. In particular, it may be worth exploring to what extent differing reasons for moving out of employment may explain this contrast, for example if young people are more likely to be leaving a job for a planned life transition (such as parenting).

In attempting to explain the remainder of the unemployment effect which does not appear to be due to income change, there has been some suggestion in existing literature that these pathways may be at least partially explained by personal debt (Jenkins et al., 2008), though there has been difficulty in establishing causality due to a lack of longitudinal studies (Richardson, Elliott, & Roberts, 2013). Similarly, it is thought that factors related to work quality (such as job insecurity and employment conditions) may play a role in mediating the link between work and MH (Rönnblad et al., 2019; Shields et al., 2021). While we were not able to explore these factors in detail in our work, it would certainly be of importance to explore in future studies.

Our study has several important strengths. Our methods make clear our assumptions regarding causal pathways and take these into account during analysis, and use of double-robust models mean that if either the exposure or the outcome model is correctly specified the results will be unbiased. We use a large, representative sample and use multiple imputations to minimise biases from missing data. However, the study does have some limitations. Our methods carry an assumption that there is no residual confounding not shown in the DAG or not perfectly measured by the variables we adjusted for, which is unlikely. In particular, our inability to incorporate measures of wealth, savings, or debt (due to these not being available in all waves of UKHLS) could make the extent of mediation by income appear smaller than it is e.g. if some individuals are able to compensate for job loss by use of savings in the short term. We also restrict our analysis only to a binary measurement of being in *v*. out of paid work, therefore not exploring other potential labour market transitions (such as moving from employment to long-term sick leave). While we selected this measure due to its appropriateness for our research question, our use of it over a more commonly used definition (e.g. categorising individuals as employed *v*. unemployed *v*. economically inactive) may reduce the generalisability of our estimates, though we note that our sensitivity analysis did not suggest this. While our focus on the short-term effects of employment is highly policy-relevant, it does not measure the effect of persistent unemployment on MH or how much this is mediated by income loss, and further work considering this would be of interest. Finally, our focus on the ATE means that our effect sizes indicate a population average effect, and acknowledge that there may be some populations for whom the effect of unemployment or degree of mediation by income may differ e.g. lone parents, or those in precarious jobs. However, our stratified analyses by gender, education, and age allow us to explore some of these potential differences while preserving sample size.

Our study has some important implications for both clinical practice and policy. Clinicians should consider job loss a potential high-risk period for MH, and consider embedding mechanisms within services to signpost affected or at-risk patients to relevant support services. From a policy perspective, our findings suggest that offering income protection to those who become unemployed will reduce around a seventh of the short-term MH consequences at a population level, and the extent will vary between groups. While these policies remain important, policymakers should also prioritise maintaining people in employment, and pursue active labour market policies where possible.

Future research using more nuanced measures of employment status and considering other labour market transitions would be of considerable interest. It would also be extremely useful to know whether the mediating role of income is more or less important when factors such as debt, wealth, job quality, hours worked, or precarity are taken into account (Rönnblad et al., 2019; Shields et al., 2021). As such, existing cohort studies should prioritise inclusion of these measures at all points when data are collected, rather than intermittently.

## Conclusions

Unemployment has a substantial detrimental effect on MH beyond its influence on people’s incomes, particularly for younger workers and those who are most disadvantaged. Preventing unemployment and moving people into work is likely to reduce the burden of poor MH at a population level, in combination with income support policies. The limited available evidence suggests that the extent of mediation by income may vary across welfare or social contexts, and further comparative research is warranted.

## Supplementary Material

Online supplement

## Figures and Tables

**Table 1:** The effect of unemployment on likelihood of CMD (direct effect blocks pathway via income change)

	Total effect (95% CI)	Direct effect (95% CI)
Odds ratio	1.66 (1.57–1.76)	1.55 (1.46–1.66)
% change in prevalence	7.09% (6.21–7.97)	6.08% (5.13–7.03)
Prevalence in unexposed	18.24% (17.87–18.61)	18.58% (18.11–19.04)
Population attributable fraction	16.49%	13.90%
% mediation	14.22% (8.04–20.40)	
	*n* = 45 497; obs = 202 297	

**Table 2:** The effect of moves into *v*. out of employment on likelihood of CMD (direct effect blocks pathway via income)

	Becoming employed		Becoming unemployed	
	Total effect (95% CI)	Direct effect (95% CI)	Total effect (95% CI)	Direct effect (95% CI)
Odds ratio	0.65 (0.58–0.72)	0.68 (0.61–0.75)	1.73 (1.61–1.85)	1.61 (1.49–1.73)
% change	−6.35% (−7.83 to −4.86)	−5.79% (−7.33 to −4.24)	7.42% (6.38–8.47)	6.30% (5.15–7.46)
Unexp prev.	29.41% (28.97–29.85)	29.62% (29.16–30.08)	16.43% (16.21–16.64)	16.51% (16.27–16.76)
PAF	−12.18%	−11.19%	18.32%	15.90%
% mediation	8.77% (0.36–17.19)		15.10% (6.81–23.39)	
	*n* = 18 353; obs = 51 224		*n* = 36 610; obs = 150 932	

PAF = population attributable fraction

**Table 3:** The effect of unemployment on likelihood of CMD, stratified by subgroups

	Sample size	Effect	Odds ratio	Unexposed prev.	Additional risk	PAF (%)	% mediated
Gender							
Men	*n* = 20 891	Total	2.02 (1.83–2.24)	15.02% (14.52–15.52)	9.11% (7.61–10.62)	23.47	14.03% (4.24–23.82)
	obs = 90 369	Direct	1.86 (1.66–2.09)	15.13% (14.44–15.82)	7.83% (6.19–9.47)	20.10	
Women	*n* = 24 634	Total	1.48 (1.38–1.58)	20.75% (20.25–21.26)	5.77% (4.69–6.85)	12.39	13.66% (4.74–22.58)
	obs = 111928	Direct	1.41 (1.30–1.52)	21.14% (20.53–21.76)	4.98% (3.81–6.15)	10.47	
Education level							
High education	*n* = 19 987	Total	1.55 (1.41–1.71)	17.42% (17.01–17.84)	6.04% (4.60–7.48)	14.94	13.60% (−0.13 to 27.33)
	obs = 82 080	Direct	1.47 (1.32–1.64)	17.51% (16.98–18.03)	5.22% (3.65–6.80)	12.89	
Med. education	*n* = 19 576	Total	1.74 (1.58–1.91)	18.07% (17.50–18.63)	7.59% (6.18–9.00)	17.42	13.66% (4.51–22.80)
	obs = 75 559	Direct	1.62 (1.46–1.79)	18.52% (17.78–19.27)	6.55% (5.06–8.05)	14.74	
Low education	*n* = 12 636	Total	1.80 (1.58–2.06)	20.50% (19.27–21.74)	8.75% (6.72–10.79)	17.75	15.25% (2.52–27.97)
	obs = 44 658	Direct	1.66 (1.44–1.91)	21.10% (19.63–22.60)	7.41% (5.30–9.53)	14.45	
Age							
Younger (25–40 years)	*n* = 20 384	Total	1.68 (1.53–1.85)	18.70% (18.22–19.18)	7.72% (6.18–9.26)	17.28	7.99% (−2.57 to 18.55)
	obs = 71777	Direct	1.62 (1.46–1.80)	18.93% (18.34–19.51)	7.10% (5.39–8.82)	15.61	
Older (41–64 years)	*n* = 29 711	Total	1.68 (1.55–1.82)	17.83% (17.31–18.36)	6.96% (5.83–8.10)	16.36	17.97% (9.95–25.99)
	obs = 130 520	Direct	1.54 (1.42–1.68)	18.26% (17.61–18.91)	5.71% (4.50–6.91)	13.16	
Poverty status							
In poverty	*n* = 18 135	Total	1.74 (1.59–1.90)	23.17% (22.08–24.25)	8.85% (7.40–10.29)	16.07	2.63% (−2.22 to 7.49)
	obs = 45 231	Direct	1.71 (1.55–1.87)	23.62% (22.42–24.81)	8.61% (7.09–10.14)	15.23	
Not in poverty	*n* = 40 023	Total	1.53 (1.41–1.66)	16.83% (16.46–17.20)	5.57% (4.42–6.71)	14.53	7.54% (−0.25 to 15.33)
	obs = 156 931	Direct	1.49 (1.37–1.62)	16.80% (16.40–17.20)	5.15% (3.97–6.33)	13.63	

PAF = population attributable fraction

## Data Availability

Original UKHLS data are held by the UK Data Service and are available on request from http://doi.org/10.5255/UKDA-SN-6614-14. The analytic code is available in an online repository from https://github.com/rachelmthomson/thomson-msm-mh.
