# Supplementary material for: To what extent does income explain the effect of unemployment on mental health? Mediation analysis in the UK Household Longitudinal Study
Source: Psychol Med. Author manuscript; Available in PMC 2023 Oct 3. (PMC10520578; doi:10.1017/S0033291722003580)

# Supplementary Material

### Table S1: Variables included within the imputation model

| Regression model | Variables | % missingness |
| --- | --- | --- |
| Linear | Log(household income)  One-year lagged log(household income)  Interaction: log(income) x gender  Interaction: log(income) x education  Interaction: log(income) x age | 2.60%  2.67%  2.60%  13.98%  2.60% |
| truncated linear  (range 0 to 100) | SF-12 physical component score (PCS)  One-year lagged SF-12 PCS  SF-12 mental component score (MCS)  One-year lagged SF-12 MCS | 12.41%  12.73%  12.41%  12.73% |
| logistic | Employment status  One-year lagged employment status  If in receipt of benefits (benefit status)  One-year lagged benefit status  Home ownership status  One-year lagged home ownership status  Marital status (coupled versus not coupled)  One-year lagged marital status  Ethnicity (White/non-White) | 5.21%  4.67%  0.48%  0.34%  1.11%  0.97%  0.13%  0.13%  1.75% |
| Ordered logit | GHQ-12 score  One-year lagged GHQ-12 score  Highest education | 11.66%  12.76%  11.67% |
| COMPLETE  (IMPUTATION VARIABLES) | Gender  Government office region  One-year lagged government office region  Number of own children in household  One-year lagged number of children  Study wave | 0%  0%  0%  0%  0%  0% |

### Table S2: Characteristics of included observations

|  | COMPLETE CASES  n = 32,138; obs = 132,962 | | IMPUTED DATA  n = 45,497; obs = 202,297 | |
| --- | --- | --- | --- | --- |
|  | Continuous variables: Mean (SD), Range | | | |
| Age | 45.36 (10.90) | 25.00 to 64.00 | 45.31 (10.93) | 25.00 to 64.00 |
| Monthly household income | £1633.34 (1707.72) | £0.04 to £201671 | £1568.82 (2015.52) | £0.04 to £202620 |
| Household income (t-1) | £1581.62 (1773.26) | £0.04 to £202620 | £1519.30 (2019.67) | £0.04 to £220830 |
| GHQ-12 score | 1.81 (3.08) | 0.00 to 12.00 | 1.88 (3.13) | 0.00 to 12.00 |
| GHQ-12 score (t-1) | 1.81 (3.05) | 0.00 to 12.00 | 1.89 (3.11) | 0.00 to 12.00 |
| Number of children | 0.71 (1.01) | 0.00 to 9.00 | 0.73 (1.05) | 0.00 to 9.00 |
| Number of children (t-1) | 0.72 (1.02) | 0.00 to 9.00 | 0.74 (1.05) | 0.00 to 9.00 |
| SF-12 physical health | 51.09 (10.14) | 4.64 to 76.29 | 50.61 (10.45) | 2.34 to 90.76 |
| SF-12 physical health (t-1) | 51.31 (10.00) | 4.48 to 76.29 | 50.85 (10.32) | 3.81 to 91.26 |
| SF-12 mental health | 48.80 (9.87) | 0.00 to 76.62 | 48.58 (10.04) | 0.00 to 80.81 |
| SF-12 mental health (t-1) | 49.07 (9.80) | 0.00 to 77.09 | 48.85 (9.98) | 0.00 to 81.89 |
|  | Categorical variables: Frequency (%) | | | |
| Gender | Male | 57,051 (42.91%) | Male | 90,369 (44.67%) |
|  | Female | 75,911 (57.09%) | Female | 111,928 (55.33%) |
| Educational attainment | High | 56,924 (42.81%) | High | 82,080 (40.57%) |
|  | Medium | 49,886 (37.52%) | Medium | 75,559 (37.35%) |
|  | Low | 26,152 (19.67%) | Low | 44,658 (22.08%) |
| Age group | Younger (25-40) | 47,023 (35.37%) | Younger (25-40) | 71,777 (35.48%) |
|  | Older (41-64) | 85,939 (64.63%) | Older 41-64) | 130,520 (64.52%) |
| Ethnicity | White | 114,778 (86.32%) | White | 166,621 (82.36%) |
|  | Non-White | 18,184 (13.68%) | Non-White | 35,676 (17.64%) |
| In employment? | Yes | 103,700 (77.99%) | Yes | 150,418 (74.35%) |
|  | No | 29,262 (22.01%) | No | 51,879 (25.65%) |
| In employment? (t-1) | Yes | 104,115 (78.30%) | Yes | 151,007 (74.65%) |
|  | No | 28,847 (21.70%) | No | 51,290 (25.35%) |
| In poverty? | Yes | 25,954 (19.52%) | Yes | 45,305 (22.40%) |
|  | No | 107,008 (80.48%) | No | 156,992 (77.60%) |
| In poverty? (t-1) | Yes | 26,231 (19.73%) | Yes | 45,830 (22.65%) |
|  | No | 106,731 (80.27%) | No | 156,467 (77.35%) |
| GHQ-12 caseness | Yes | 25,185 (18.94%) | Yes | 40,164 (19.85%) |
|  | No | 107,777 (81.06%) | No | 162,133 (80.15%) |
| GHQ-12 caseness (t-1) | Yes | 25,102 (18.88%) | Yes | 40,177 (19.86%) |
|  | No | 107,860 (81.12%) | No | 162,120 (80.14%) |
| Receives benefits? | Yes | 83,508 (62.81%) | Yes | 131,899 (65.20%) |
|  | No | 49,454 (37.19%) | No | 70,398 (34.80%) |
| Receives benefits? (t-1) | Yes | 83,335 (62.68%) | Yes | 131,560 (65.03%) |
|  | No | 49,627 (37.32%) | No | 70,737 (34.97%) |
| Owns home? | Yes | 98,228 (73.88%) | Yes | 143,339 (70.86%) |
|  | No | 34,734 (26.12%) | No | 58,958 (29.14%) |
| Owns home? (t-1) | Yes | 97,760 (73.52%) | Yes | 142,772 (70.58%) |
|  | No | 35,202 (26.48%) | No | 59,525 (29.42%) |
| Has partner? | Yes | 99,408 (74.76%) | Yes | 149,911 (74.10%) |
|  | No | 33,554 (25.24%) | No | 52,387 (25.90%) |
| Has partner? (t-1) | Yes | 99,027 (74.48%) | Yes | 149,408 (73.86%) |
|  | No | 33,935 (25.52%) | No | 52,889 (26.14%) |
| Govt. office region | North East | 5,548 (4.17%) | North East | 7,596 (3.75%) |
|  | North West | 14,495 (10.90%) | North West | 20,490 (10.13%) |
|  | Yorkshire & the Humber | 11,152 (8.39%) | Yorkshire & the Humber | 16,681 (8.25%) |
|  | East Midlands | 10,398 (7.82%) | East Midlands | 15,057 (7.44%) |
|  | West Midlands | 11,359 (8.54%) | West Midlands | 16,538 (8.18%) |
|  | East of England | 12,281 (9.24%) | East of England | 17,214 (8.51%) |
|  | London | 14,326 (10.77%) | London | 26,806 (13.25%) |
|  | South East | 17,150 (12.90%) | South East | 23,884 (11.81%) |
|  | South West | 12,041 (9.06%) | South West | 15,917 (7.87%) |
|  | Wales | 7,507 (5.65%) | Wales | 12,887 (6.37%) |
|  | Scotland | 10,833 (8.15%) | Scotland | 16,846 (8.33%) |
|  | Northern Ireland | 5,872 (4.42%) | Northern Ireland | 12,381 (6.12%) |
| Govt. office region (t-1) | North East | 5,541 (4.17%) | North East | 7,590 (3.75%) |
|  | North West | 14,500 (10.91%) | North West | 20,488 (10.13%) |
|  | Yorkshire & the Humber | 11,142 (8.38%) | Yorkshire & the Humber | 16,669 (8.24%) |
|  | East Midlands | 10,380 (7.81%) | East Midlands | 15,042 (7.44%) |
|  | West Midlands | 11,334 (8.52%) | West Midlands | 16,523 (8.17%) |
|  | East of England | 12,254 (9.22%) | East of England | 17,163 (8.48%) |
|  | London | 14,432 (10.85%) | London | 26,999 (13.35%) |
|  | South East | 17,174 (12.92%) | South East | 23,860 (11.79%) |
|  | South West | 12,001 (9.03%) | South West | 15,870 (7.84%) |
|  | Wales | 7,513 (5.65%) | Wales | 12,887 (6.37%) |
|  | Scotland | 10,820 (8.14%) | Scotland | 16,822 (8.32%) |
|  | Northern Ireland | 5,871 (4.42%) | Northern Ireland | 12,384 (6.12%) |

### Table S3: Characteristics of individuals experiencing employment transitions of interest

|  | BECOMING UNEMPLOYED  n = 7,984; obs = 8,839 | | BECOMING EMPLOYED  n = 7,399; obs = 8,250 | |
| --- | --- | --- | --- | --- |
|  | Continuous variables: Mean (SD), Range | | | |
| Age | 47.41 (12.00) | 25.00 to 64.00 | 42.77 (11.32) | 25.00 to 64.00 |
| Monthly household income | £1314.46 (1772.55) | £0.08 to £95166 | £1395.29 (1785.78) | £0.52 to £87653 |
| Household income (t-1) | £1609.66 (1605.79) | £1.11 to £55409 | £1091.22 (1588.66) | £0.04 to 95166 |
| GHQ-12 score | 2.54 (3.63) | 0.00 to 12.00 | 1.73 (2.98) | 0.00 to 12.00 |
| GHQ-12 score (t-1) | 2.15 (3.32) | 0.00 to 12.00 | 2.43 (3.44) | 0.00 to 12.00 |
| Number of children | 0.65 (1.04) | 0.00 to 7.00 | 0.88 (1.15) | 0.00 to 7.00 |
| Number of children (t-1) | 0.64 (1.03) | 0.00 to 7.00 | 0.91 (1.16) | 0.00 to 7.00 |
| SF-12 physical health | 49.38 (11.18) | 7.15 to 80.09 | 51.00 (9.59) | 4.64 to 82.50 |
| SF-12 physical health (t-1) | 49.95 (10.54) | 4.70 to 80.67 | 51.10 (10.07) | 7.40 to 82.73 |
| SF-12 mental health | 47.74 (11.03) | 2.42 to 75.45 | 48.90 (9.76) | 0.45 to 76.03 |
| SF-12 mental health (t-1) | 48.44 (10.31) | 0.19 to 75.91 | 47.73 (10.58) | 1.98 to 75.98 |
|  | Categorical variables: Frequency (%) | | | |
| Gender | Male | 5,245 (59.33%) | Male | 5,002 (60.62%) |
|  | Female | 3,595 (40.67%) | Female | 3,249 (39.38%) |
| Educational attainment | High | 3,573 (40.43%) | High | 3,369 (40.83%) |
|  | Medium | 3,298 (37.31%) | Medium | 3,111 (37.70%) |
|  | Low | 1,968 (22.26%) | Low | 1,771 (21.47%) |
| Age group | Younger (25-40) | 2,852 (32.27%) | Younger (25-40) | 3,781 (45.83%) |
|  | Older (41-64) | 5,987 (67.73%) | Older 41-64) | 4,469 (54.17%) |
| Ethnicity | White | 7,111 (80.45%) | White | 6,122 (74.21%) |
|  | Non-White | 1,728 (19.55%) | Non-White | 2,128 (25.79%) |
| In poverty? | Yes | 3,298 (37.31%) | Yes | 2,485 (30.11%) |
|  | No | 5,542 (62.69%) | No | 5,766 (69.89%) |
| In poverty? (t-1) | Yes | 1,977 (22.36%) | Yes | 3,773 (45.73%) |
|  | No | 6,863 (77.64%) | No | 4,477 (54.27%) |
| GHQ-12 caseness | Yes | 2,418 (27.36%) | Yes | 1,501 (18.19%) |
|  | No | 6,421 (72.64%) | No | 6,749 (81.81%) |
| GHQ-12 caseness (t-1) | Yes | 2,003 (22.66%) | Yes | 2,167 (26.27%) |
|  | No | 6,837 (77.34%) | No | 6,083 (73.73%) |
| Receives benefits? | Yes | 6,275 (70.99%) | Yes | 5,831 (70.67%) |
|  | No | 2,564 (29.01%) | No | 2,420 (29.33%) |
| Receives benefits? (t-1) | Yes | 5,477 (61.96%) | Yes | 6,361 (77.10%) |
|  | No | 3,362 (38.04%) | No | 1,889 (22.90%) |
| Owns home? | Yes | 6,171 (69.81%) | Yes | 4,869 (59.01%) |
|  | No | 2,669 (30.19%) | No | 3,381 (40.99%) |
| Owns home? (t-1) | Yes | 6,184 (69.96%) | Yes | 4,872 (59.05%) |
|  | No | 2,655 (30.04%) | No | 3,379 (40.95%) |
| Has partner? | Yes | 6,447 (72.93%) | Yes | 5,639 (68.35%) |
|  | No | 2,392 (27.07%) | No | 2,611 (31.65%) |
| Has partner? (t-1) | Yes | 6,481 (73.33%) | Yes | 5,607 (67.96%) |
|  | No | 2,358 (26.67%) | No | 2,643 (32.04%) |
| Govt. office region | North East | 317 (3.58%) | North East | 260 (3.15%) |
|  | North West | 854 (9.66%) | North West | 767 (9.30%) |
|  | Yorkshire & the Humber | 710 (8.04%) | Yorkshire & the Humber | 656 (7.95%) |
|  | East Midlands | 627 (7.09%) | East Midlands | 583 (7.07%) |
|  | West Midlands | 745 (8.42%) | West Midlands | 703 (8.52%) |
|  | East of England | 763 (8.63%) | East of England | 690 (8.37%) |
|  | London | 1,318 (14.91%) | London | 1,554 (18.83%) |
|  | South East | 1,089 (12.32%) | South East | 963 (11.67%) |
|  | South West | 657 (7.43%) | South West | 615 (7.46%) |
|  | Wales | 583 (6.59%) | Wales | 503 (6.09%) |
|  | Scotland | 670 (7.58%) | Scotland | 533 (6.46%) |
|  | Northern Ireland | 508 (5.75%) | Northern Ireland | 424 (5.13%) |
| Govt. office region (t-1) | North East | 311 (3.52%) | North East | 265 (3.21%) |
|  | North West | 854 (9.66%) | North West | 762 (9.24%) |
|  | Yorkshire & the Humber | 701 (7.93%) | Yorkshire & the Humber | 652 (7.9%) |
|  | East Midlands | 618 (6.99%) | East Midlands | 586 (7.1%) |
|  | West Midlands | 737 (8.34%) | West Midlands | 703 (8.53%) |
|  | East of England | 767 (8.68%) | East of England | 686 (8.32%) |
|  | London | 1,344 (15.21%) | London | 1,572 (19.06%) |
|  | South East | 1,107 (12.53%) | South East | 957 (11.6%) |
|  | South West | 649 (7.34%) | South West | 610 (7.39%) |
|  | Wales | 573 (6.48%) | Wales | 507 (6.14%) |
|  | Scotland | 671 (7.59%) | Scotland | 526 (6.38%) |
|  | Northern Ireland | 507 (5.74%) | Northern Ireland | 425 (5.15%) |

### Table S4: Standardised mean differences of confounding variables between exposed and unexposed groups in primary and stratified analyses of **total effect** of unemployment on mental health, before and after application of inverse probability of treatment weights (IPTWs)

|  | Whole imputed sample | | Complete cases | | Into unemployment | | Into work | | Men | | Women | |
| --- | --- | --- | --- | --- | --- | --- | --- | --- | --- | --- | --- | --- |
|  | Pre-IPTW | Post-IPTW | Pre-IPTW | Post-IPTW | Pre-IPTW | Post-IPTW | Pre-IPTW | Post-IPTW | Pre-IPTW | Post-IPTW | Pre-IPTW | Post-IPTW |
| 1. Gender | -0.295 | -0.014 | -0.286 | -0.030 | -0.166 | -0.013 | 0.141 | 0.007 | . | . | . | . |
| 1. Educ: Medium | 0.035 | -0.029 | 0.077 | -0.028 | 0.009 | 0.001 | -0.024 | 0.004 | -0.023 | -0.003 | 0.083 | -0.041 |
| 1. Educ: Low | 0.370 | -0.016 | 0.290 | -0.015 | 0.112 | 0.009 | -0.332 | 0.005 | 0.363 | -0.010 | 0.393 | -0.011 |
| 1. Ethnicity | 0.182 | 0.029 | 0.093 | 0.026 | 0.114 | 0.025 | 0.049 | 0.036 | 0.038 | 0.081 | 0.264 | 0.007 |
| 1. Age | 0.369 | -0.025 | 0.422 | -0.027 | 0.275 | 0.021 | -0.499 | 0.030 | 0.532 | -0.012 | 0.296 | -0.034 |
| 1. Age squared | 0.413 | -0.019 | 0.470 | -0.020 | 0.317 | 0.021 | -0.517 | 0.033 | 0.578 | -0.008 | 0.339 | -0.029 |
| 1. Prev unemployed? | 2.493 | 0.009 | 2.460 | 0.010 | . | . | . | . | 2.348 | 0.011 | 2.539 | 0.005 |
| 1. No. children | -0.024 | -0.035 | -0.030 | -0.035 | -0.100 | -0.030 | 0.141 | 0.011 | -0.360 | -0.016 | 0.135 | -0.020 |
| 1. Partner? | -0.224 | -0.043 | -0.199 | -0.031 | -0.083 | -0.044 | 0.063 | 0.011 | -0.402 | -0.042 | -0.097 | -0.016 |
| 1. Receive benefits? | 0.506 | -0.028 | 0.484 | -0.010 | 0.076 | -0.021 | -0.222 | -0.012 | 0.413 | -0.022 | 0.544 | -0.027 |
| 1. Home owner? | -0.452 | -0.035 | -0.395 | -0.030 | -0.158 | -0.035 | 0.144 | 0.023 | -0.480 | -0.056 | -0.432 | -0.014 |
| 1. North West | 0.025 | -0.018 | 0.038 | -0.029 | -0.011 | -0.012 | -0.055 | 0.000 | 0.030 | -0.033 | 0.022 | -0.009 |
| 1. Yorkshire | 0.038 | -0.013 | 0.049 | -0.020 | -0.002 | -0.002 | -0.049 | 0.006 | 0.026 | -0.019 | 0.048 | -0.011 |
| 1. East Midlands | -0.018 | -0.009 | -0.008 | -0.010 | -0.023 | -0.003 | 0.000 | 0.001 | -0.003 | -0.021 | -0.024 | -0.002 |
| 1. West Midlands | 0.032 | 0.000 | 0.037 | 0.007 | 0.016 | -0.002 | -0.014 | -0.002 | 0.013 | 0.020 | 0.041 | -0.005 |
| 1. East of England | -0.045 | -0.002 | -0.034 | -0.005 | -0.005 | -0.006 | 0.037 | 0.001 | -0.057 | -0.016 | -0.037 | 0.012 |
| 1. London | 0.062 | 0.049 | 0.003 | 0.047 | 0.080 | 0.034 | 0.112 | 0.036 | 0.011 | 0.070 | 0.091 | 0.040 |
| 1. South East | -0.084 | 0.020 | -0.051 | 0.021 | 0.000 | 0.023 | 0.076 | 0.025 | -0.099 | 0.018 | -0.079 | 0.019 |
| 1. South West | -0.061 | -0.009 | -0.032 | -0.005 | -0.036 | -0.011 | 0.034 | -0.016 | -0.044 | -0.024 | -0.069 | -0.001 |
| 1. Wales | 0.018 | -0.006 | -0.010 | 0.006 | 0.009 | -0.010 | -0.025 | -0.008 | 0.024 | 0.006 | 0.014 | -0.010 |
| 1. Scotland | -0.028 | -0.015 | -0.022 | -0.016 | -0.038 | -0.015 | -0.054 | -0.019 | 0.000 | -0.010 | -0.048 | -0.023 |
| 1. Northern Ireland | 0.046 | -0.018 | 0.006 | -0.015 | -0.006 | -0.017 | -0.086 | -0.029 | 0.085 | -0.020 | 0.021 | -0.023 |
| 1. Prev GHQ case? | 0.331 | 0.035 | 0.325 | 0.042 | 0.176 | 0.005 | -0.119 | -0.037 | 0.368 | 0.050 | 0.285 | 0.018 |
| 1. SF12 Phys. Health | -0.712 | 0.002 | -0.685 | -0.002 | -0.319 | -0.002 | 0.597 | 0.027 | -0.777 | 0.015 | -0.671 | -0.004 |
| 1. SF12 Ment. Health | -0.364 | -0.020 | -0.352 | -0.025 | -0.158 | -0.004 | 0.197 | 0.025 | -0.394 | -0.014 | -0.312 | -0.015 |
| 1. Prev. poverty | 0.576 | 0.038 | 0.558 | 0.046 | 0.203 | 0.019 | 0.012 | 0.008 | 0.548 | 0.073 | 0.593 | 0.020 |
| 1. Prev HH income | -0.585 | -0.030 | -0.545 | -0.035 | -0.152 | -0.004 | -0.034 | -0.009 | -0.564 | -0.064 | -0.598 | -0.011 |

|  | High education | | Medium education | | Low education | | Younger working-age | | Older working-age | | In poverty only | | Not in poverty only | |
| --- | --- | --- | --- | --- | --- | --- | --- | --- | --- | --- | --- | --- | --- | --- |
|  | Pre-IPTW | Post-IPTW | Pre-IPTW | Post-IPTW | Pre-IPTW | Post-IPTW | Pre-IPTW | Post-IPTW | Pre-IPTW | Post-IPTW | Pre-IPTW | Post-IPTW | Pre-IPTW | Post-IPTW |
| 1. | -0.240 | -0.016 | -0.364 | 0.004 | -0.352 | -0.009 | -0.475 | -0.024 | -0.225 | 0.008 | -0.310 | -0.002 | -0.293 | -0.034 |
| 2. | . | . | . | . | . | . | 0.203 | -0.053 | -0.042 | -0.011 | -0.051 | -0.009 | 0.034 | -0.029 |
| 3. | . | . | . | . | . | . | 0.310 | -0.037 | 0.374 | -0.006 | 0.348 | 0.018 | 0.303 | -0.042 |
| 4. | 0.129 | 0.047 | 0.256 | 0.029 | 0.263 | -0.021 | 0.351 | 0.027 | 0.122 | 0.018 | 0.013 | 0.005 | 0.109 | 0.046 |
| 5. | 0.472 | -0.059 | 0.203 | -0.008 | 0.296 | 0.009 | -0.094 | -0.046 | 0.599 | 0.021 | 0.267 | 0.021 | 0.608 | -0.063 |
| 6. | 0.521 | -0.053 | 0.248 | -0.004 | 0.331 | 0.010 | -0.092 | -0.044 | 0.616 | 0.020 | 0.302 | 0.021 | 0.658 | -0.056 |
| 7. | 2.040 | 0.001 | 2.526 | 0.009 | 2.905 | 0.012 | 2.284 | -0.005 | 2.612 | 0.009 | 2.229 | 0.008 | 2.464 | -0.002 |
| 8. | -0.076 | -0.052 | 0.083 | -0.027 | -0.072 | -0.028 | 0.463 | -0.080 | -0.260 | -0.002 | -0.137 | -0.017 | -0.211 | -0.031 |
| 9. | -0.124 | -0.078 | -0.191 | -0.030 | -0.330 | -0.021 | -0.215 | -0.057 | -0.246 | -0.026 | -0.238 | -0.028 | -0.120 | -0.049 |
| 10. | 0.309 | -0.024 | 0.486 | -0.021 | 0.628 | -0.021 | 0.631 | -0.063 | 0.480 | -0.021 | 0.264 | 0.013 | 0.435 | -0.038 |
| 11. | -0.202 | -0.060 | -0.442 | -0.033 | -0.564 | -0.032 | -0.590 | -0.017 | -0.453 | -0.038 | -0.220 | -0.043 | -0.207 | -0.034 |
| 12. | 0.022 | -0.027 | 0.003 | -0.017 | 0.073 | -0.009 | 0.030 | -0.045 | 0.028 | 0.000 | 0.052 | 0.000 | 0.016 | -0.023 |
| 13. | 0.027 | -0.017 | 0.016 | -0.014 | 0.047 | 0.000 | 0.046 | -0.017 | 0.039 | -0.013 | 0.034 | 0.003 | 0.032 | -0.014 |
| 14. | 0.022 | -0.019 | -0.067 | 0.002 | -0.028 | 0.005 | -0.031 | -0.023 | -0.014 | 0.002 | 0.002 | -0.005 | -0.011 | -0.014 |
| 15. | 0.013 | -0.005 | 0.014 | 0.007 | 0.061 | -0.003 | 0.055 | 0.004 | 0.023 | -0.003 | 0.039 | 0.002 | 0.022 | -0.009 |
| 16. | -0.011 | -0.012 | -0.043 | -0.006 | -0.063 | 0.020 | -0.020 | 0.006 | -0.057 | -0.010 | -0.053 | 0.001 | -0.035 | -0.009 |
| 17. | 0.039 | 0.069 | 0.141 | 0.040 | 0.153 | -0.005 | 0.134 | 0.057 | 0.036 | 0.034 | -0.028 | -0.001 | 0.015 | 0.074 |
| 18. | -0.014 | 0.024 | -0.065 | 0.011 | -0.105 | -0.005 | -0.102 | 0.021 | -0.080 | 0.022 | -0.113 | -0.012 | -0.050 | 0.031 |
| 19. | -0.038 | -0.013 | -0.072 | -0.007 | -0.037 | 0.001 | -0.077 | -0.025 | -0.061 | 0.001 | -0.078 | -0.009 | -0.039 | -0.004 |
| 20. | -0.027 | -0.007 | 0.037 | 0.006 | -0.063 | -0.008 | -0.015 | 0.009 | 0.032 | -0.010 | 0.021 | -0.006 | 0.025 | -0.013 |
| 21. | -0.028 | -0.017 | -0.017 | -0.012 | -0.105 | -0.011 | -0.062 | 0.003 | -0.023 | -0.023 | 0.058 | 0.017 | -0.024 | -0.016 |
| 22. | -0.013 | -0.039 | 0.026 | -0.004 | 0.028 | 0.008 | -0.025 | -0.010 | 0.072 | -0.019 | 0.080 | 0.008 | 0.049 | -0.022 |
| 23. | 0.198 | 0.036 | 0.361 | 0.035 | 0.408 | 0.030 | 0.309 | 0.040 | 0.344 | 0.028 | 0.329 | 0.006 | 0.282 | 0.046 |
| 24. | -0.555 | 0.022 | -0.628 | -0.007 | -0.827 | -0.019 | -0.495 | 0.018 | -0.785 | -0.003 | -0.591 | -0.030 | -0.729 | 0.031 |
| 25. | -0.169 | -0.030 | -0.407 | -0.010 | -0.479 | -0.018 | -0.400 | -0.015 | -0.365 | -0.015 | -0.372 | -0.016 | -0.279 | -0.025 |
| 26. | 0.416 | 0.049 | 0.547 | 0.049 | 0.614 | 0.024 | 0.797 | 0.030 | 0.512 | 0.038 | 0.246 | 0.024 | 0.296 | 0.097 |
| 27. | -0.394 | -0.046 | -0.537 | -0.043 | -0.651 | -0.009 | -0.813 | -0.025 | -0.535 | -0.025 | -0.262 | -0.027 | -0.334 | -0.052 |

Highlighted cells are those with standardised mean difference ≥ 0.1, indicating a greater than negligible statistical difference between exposed and unexposed groups

### Table S5: Standardised mean differences of confounding variables between exposed and unexposed groups in primary and stratified analyses of **direct effect** of unemployment on mental health, before and after application of inverse probability of treatment weights (IPTWs)

|  | | | Whole imputed sample | | | | Complete cases | | | | Into unemployment | | | | Into work | | | | Men | | | | Women | | | |
| --- | --- | --- | --- | --- | --- | --- | --- | --- | --- | --- | --- | --- | --- | --- | --- | --- | --- | --- | --- | --- | --- | --- | --- | --- | --- | --- |
|  | | | Pre-IPTW | | Post-IPTW | | Pre-IPTW | | Post-IPTW | | Pre-IPTW | | Post-IPTW | | Pre-IPTW | | Post-IPTW | | Pre-IPTW | | Post-IPTW | | Pre-IPTW | | Post-IPTW | |
| 1. Gender | | | -0.295 | | -0.037 | | -0.286 | | -0.069 | | -0.166 | | -0.049 | | 0.141 | | 0.008 | | . | | . | | . | | . | |
| 1. Educ: Medium | | | 0.035 | | -0.024 | | 0.077 | | -0.025 | | 0.009 | | 0.011 | | -0.024 | | 0.002 | | -0.023 | | 0.005 | | 0.083 | | -0.037 | |
| 1. Educ: Low | | | 0.370 | | -0.031 | | 0.290 | | -0.038 | | 0.112 | | -0.004 | | -0.332 | | 0.015 | | 0.363 | | -0.048 | | 0.393 | | -0.013 | |
| 1. Ethnicity | | | 0.182 | | 0.044 | | 0.093 | | 0.058 | | 0.114 | | 0.081 | | 0.049 | | 0.051 | | 0.038 | | 0.106 | | 0.264 | | 0.014 | |
| 1. Age | | | 0.369 | | -0.028 | | 0.422 | | -0.041 | | 0.275 | | 0.007 | | -0.499 | | 0.040 | | 0.532 | | -0.014 | | 0.296 | | -0.033 | |
| 1. Age squared | | | 0.413 | | -0.023 | | 0.470 | | -0.034 | | 0.317 | | 0.007 | | -0.517 | | 0.041 | | 0.578 | | -0.009 | | 0.339 | | -0.030 | |
| 1. Prev unemployed? | | | 2.493 | | -0.023 | | 2.460 | | -0.030 | | . | | . | | . | | . | | 2.348 | | -0.036 | | 2.539 | | -0.022 | |
| 1. No. children | | | -0.024 | | -0.009 | | -0.030 | | 0.007 | | -0.100 | | 0.027 | | 0.141 | | 0.017 | | -0.360 | | 0.019 | | 0.135 | | -0.007 | |
| 1. Partner? | | | -0.224 | | -0.031 | | -0.199 | | -0.018 | | -0.083 | | -0.032 | | 0.063 | | 0.016 | | -0.402 | | -0.019 | | -0.097 | | -0.013 | |
| 1. Receive benefits? | | | 0.506 | | -0.022 | | 0.484 | | 0.000 | | 0.076 | | 0.009 | | -0.222 | | -0.005 | | 0.413 | | -0.031 | | 0.544 | | -0.018 | |
| 1. Home owner? | | | -0.452 | | -0.033 | | -0.395 | | -0.025 | | -0.158 | | -0.074 | | 0.144 | | 0.011 | | -0.480 | | -0.066 | | -0.432 | | -0.007 | |
| 1. North West | | | 0.025 | | -0.020 | | 0.038 | | -0.030 | | -0.011 | | -0.024 | | -0.055 | | -0.011 | | 0.030 | | -0.043 | | 0.022 | | -0.008 | |
| 1. Yorkshire | | | 0.038 | | -0.014 | | 0.049 | | -0.026 | | -0.002 | | -0.004 | | -0.049 | | 0.002 | | 0.026 | | -0.027 | | 0.048 | | -0.007 | |
| 1. East Midlands | | | -0.018 | | -0.015 | | -0.008 | | -0.032 | | -0.023 | | -0.015 | | 0.000 | | 0.000 | | -0.003 | | -0.043 | | -0.024 | | 0.001 | |
| 1. West Midlands | | | 0.032 | | -0.006 | | 0.037 | | -0.006 | | 0.016 | | -0.008 | | -0.014 | | 0.003 | | 0.013 | | 0.003 | | 0.041 | | -0.002 | |
| 1. East of England | | | -0.045 | | -0.011 | | -0.034 | | -0.016 | | -0.005 | | -0.023 | | 0.037 | | -0.004 | | -0.057 | | -0.030 | | -0.037 | | 0.009 | |
| 1. London | | | 0.062 | | 0.061 | | 0.003 | | 0.072 | | 0.080 | | 0.080 | | 0.112 | | 0.056 | | 0.011 | | 0.094 | | 0.091 | | 0.041 | |
| 1. South East | | | -0.084 | | 0.025 | | -0.051 | | 0.020 | | 0.000 | | 0.028 | | 0.076 | | 0.024 | | -0.099 | | 0.037 | | -0.079 | | 0.019 | |
| 1. South West | | | -0.061 | | 0.000 | | -0.032 | | 0.009 | | -0.036 | | 0.002 | | 0.034 | | -0.014 | | -0.044 | | -0.015 | | -0.069 | | 0.006 | |
| 1. Wales | | | 0.018 | | -0.015 | | -0.010 | | -0.001 | | 0.009 | | -0.019 | | -0.025 | | 0.001 | | 0.024 | | 0.000 | | 0.014 | | -0.018 | |
| 1. Scotland | | | -0.028 | | -0.010 | | -0.022 | | 0.002 | | -0.038 | | -0.021 | | -0.054 | | -0.039 | | 0.000 | | 0.011 | | -0.048 | | -0.027 | |
| 1. Northern Ireland | | | 0.046 | | -0.024 | | 0.006 | | -0.015 | | -0.006 | | -0.023 | | -0.086 | | -0.018 | | 0.085 | | -0.029 | | 0.021 | | -0.029 | |
| 1. Prev GHQ case? | | | 0.331 | | 0.014 | | 0.325 | | 0.005 | | 0.176 | | -0.002 | | -0.119 | | -0.028 | | 0.368 | | 0.019 | | 0.285 | | 0.007 | |
| 1. SF12 Phys. Health | | | -0.712 | | 0.022 | | -0.685 | | 0.026 | | -0.319 | | 0.001 | | 0.597 | | 0.006 | | -0.777 | | 0.051 | | -0.671 | | 0.013 | |
| 1. SF12 Ment. Health | | | -0.364 | | -0.009 | | -0.352 | | -0.009 | | -0.158 | | -0.007 | | 0.197 | | 0.021 | | -0.394 | | 0.001 | | -0.312 | | -0.007 | |
| 1. Prev. poverty | | | 0.576 | | 0.073 | | 0.558 | | 0.087 | | 0.203 | | 0.121 | | 0.012 | | -0.009 | | 0.548 | | 0.122 | | 0.593 | | 0.045 | |
| 1. Poverty | | | 0.627 | | -0.022 | | 0.619 | | -0.032 | | 0.537 | | 0.023 | | -0.283 | | 0.088 | | 0.621 | | -0.012 | | 0.633 | | -0.036 | |
| 1. HH income | | | -0.660 | | 0.083 | | -0.628 | | 0.119 | | -0.534 | | 0.032 | | 0.300 | | -0.116 | | -0.658 | | 0.104 | | -0.663 | | 0.079 | |
| 1. Prev HH income | | | -0.585 | | -0.043 | | -0.545 | | -0.048 | | -0.152 | | -0.106 | | -0.034 | | -0.012 | | -0.564 | | -0.089 | | -0.598 | | -0.020 | |
|  | High education | | | Medium education | | | | Low education | | | | Younger working-age | | | | Older working-age | | | | In poverty only | | | | Not in poverty only | | |
|  | Pre-IPTW | Post-IPTW | | Pre-IPTW | | Post-IPTW | | Pre-IPTW | | Post-IPTW | | Pre-IPTW | | Post-IPTW | | Pre-IPTW | | Post-IPTW | | Pre-IPTW | | Post-IPTW | | Pre-IPTW | | Post-IPTW |
| 1. | -0.240 | -0.033 | | -0.364 | | -0.017 | | -0.352 | | -0.052 | | -0.475 | | -0.038 | | -0.225 | | -0.020 | | -0.310 | | -0.013 | | -0.293 | | -0.043 |
| 2. | . | . | | . | | . | | . | | . | | 0.203 | | -0.050 | | -0.042 | | -0.003 | | -0.051 | | -0.012 | | 0.034 | | -0.032 |
| 3. | . | . | | . | | . | | . | | . | | 0.310 | | -0.048 | | 0.374 | | -0.026 | | 0.348 | | 0.018 | | 0.303 | | -0.045 |
| 4. | 0.129 | 0.064 | | 0.256 | | 0.044 | | 0.263 | | -0.014 | | 0.351 | | 0.039 | | 0.122 | | 0.036 | | 0.013 | | 0.008 | | 0.109 | | 0.060 |
| 5. | 0.472 | -0.065 | | 0.203 | | -0.004 | | 0.296 | | 0.005 | | -0.094 | | -0.041 | | 0.599 | | 0.011 | | 0.267 | | 0.015 | | 0.608 | | -0.062 |
| 6. | 0.521 | -0.060 | | 0.248 | | -0.001 | | 0.331 | | 0.007 | | -0.092 | | -0.037 | | 0.616 | | 0.010 | | 0.302 | | 0.015 | | 0.658 | | -0.055 |
| 7. | 2.040 | -0.024 | | 2.526 | | -0.022 | | 2.905 | | -0.027 | | 2.284 | | -0.021 | | 2.612 | | -0.034 | | 2.229 | | -0.005 | | 2.464 | | -0.009 |
| 8. | -0.076 | -0.015 | | 0.083 | | -0.016 | | -0.072 | | 0.010 | | 0.463 | | -0.054 | | -0.260 | | 0.029 | | -0.137 | | -0.015 | | -0.211 | | -0.005 |
| 9. | -0.124 | -0.070 | | -0.191 | | -0.019 | | -0.330 | | -0.002 | | -0.215 | | -0.062 | | -0.246 | | 0.001 | | -0.238 | | -0.016 | | -0.120 | | -0.061 |
| 10. | 0.309 | -0.015 | | 0.486 | | -0.015 | | 0.628 | | -0.010 | | 0.631 | | -0.042 | | 0.480 | | -0.021 | | 0.264 | | 0.013 | | 0.435 | | -0.031 |
| 11. | -0.202 | -0.075 | | -0.442 | | -0.031 | | -0.564 | | -0.011 | | -0.590 | | -0.020 | | -0.453 | | -0.027 | | -0.220 | | -0.041 | | -0.207 | | -0.055 |
| 12. | 0.022 | -0.037 | | 0.003 | | -0.010 | | 0.073 | | -0.004 | | 0.030 | | -0.054 | | 0.028 | | -0.001 | | 0.052 | | 0.003 | | 0.016 | | -0.036 |
| 13. | 0.027 | -0.025 | | 0.016 | | -0.001 | | 0.047 | | -0.014 | | 0.046 | | -0.013 | | 0.039 | | -0.020 | | 0.034 | | -0.003 | | 0.032 | | -0.022 |
| 14. | 0.022 | -0.017 | | -0.067 | | -0.016 | | -0.028 | | 0.012 | | -0.031 | | -0.036 | | -0.014 | | 0.003 | | 0.002 | | -0.003 | | -0.011 | | -0.015 |
| 15. | 0.013 | -0.008 | | 0.014 | | -0.010 | | 0.061 | | 0.003 | | 0.055 | | 0.003 | | 0.023 | | -0.012 | | 0.039 | | 0.002 | | 0.022 | | -0.009 |
| 16. | -0.011 | -0.027 | | -0.043 | | -0.008 | | -0.063 | | 0.016 | | -0.020 | | -0.003 | | -0.057 | | -0.016 | | -0.053 | | -0.002 | | -0.035 | | -0.012 |
| 17. | 0.039 | 0.088 | | 0.141 | | 0.046 | | 0.153 | | -0.009 | | 0.134 | | 0.077 | | 0.036 | | 0.040 | | -0.028 | | 0.003 | | 0.015 | | 0.092 |
| 18. | -0.014 | 0.016 | | -0.065 | | 0.021 | | -0.105 | | 0.012 | | -0.102 | | 0.026 | | -0.080 | | 0.029 | | -0.113 | | -0.007 | | -0.050 | | 0.027 |
| 19. | -0.038 | 0.006 | | -0.072 | | -0.010 | | -0.037 | | 0.006 | | -0.077 | | -0.016 | | -0.061 | | 0.009 | | -0.078 | | -0.007 | | -0.039 | | -0.001 |
| 20. | -0.027 | -0.010 | | 0.037 | | -0.008 | | -0.063 | | -0.013 | | -0.015 | | -0.007 | | 0.032 | | -0.013 | | 0.021 | | -0.010 | | 0.025 | | -0.017 |
| 21. | -0.028 | -0.009 | | -0.017 | | -0.003 | | -0.105 | | -0.009 | | -0.062 | | 0.010 | | -0.023 | | -0.020 | | 0.058 | | 0.016 | | -0.024 | | -0.006 |
| 22. | -0.013 | -0.042 | | 0.026 | | -0.006 | | 0.028 | | -0.006 | | -0.025 | | -0.018 | | 0.072 | | -0.024 | | 0.080 | | 0.000 | | 0.049 | | -0.030 |
| 23. | 0.198 | 0.006 | | 0.361 | | 0.026 | | 0.408 | | 0.012 | | 0.309 | | 0.022 | | 0.344 | | 0.004 | | 0.329 | | -0.002 | | 0.282 | | 0.031 |
| 24. | -0.555 | 0.029 | | -0.628 | | 0.022 | | -0.827 | | 0.000 | | -0.495 | | 0.029 | | -0.785 | | 0.023 | | -0.591 | | -0.025 | | -0.729 | | 0.033 |
| 25. | -0.169 | -0.022 | | -0.407 | | -0.002 | | -0.479 | | -0.005 | | -0.400 | | -0.014 | | -0.365 | | 0.008 | | -0.372 | | -0.015 | | -0.279 | | -0.010 |
| 26. | 0.416 | 0.107 | | 0.547 | | 0.075 | | 0.614 | | 0.055 | | 0.797 | | 0.075 | | 0.512 | | 0.060 | | 0.246 | | 0.032 | | 0.296 | | 0.137 |
| 27. | 0.493 | -0.006 | | 0.596 | | -0.015 | | 0.650 | | -0.050 | | 0.881 | | 0.003 | | 0.553 | | -0.044 | | . | | . | | . | | . |
| 28. | -0.497 | 0.076 | | -0.607 | | 0.072 | | -0.708 | | 0.115 | | -0.910 | | 0.059 | | -0.604 | | 0.113 | | -0.244 | | 0.085 | | -0.351 | | 0.123 |
| 29. | -0.394 | -0.069 | | -0.537 | | -0.052 | | -0.651 | | -0.018 | | -0.813 | | -0.035 | | -0.535 | | -0.035 | | -0.262 | | -0.030 | | -0.334 | | -0.095 |

*Highlighted cells are those with standardised mean difference ≥ 0.1, indicating a greater than negligible statistical difference between exposed and unexposed groups*

### Table S6: The effect of unemployment on likelihood of common mental disorder in complete cases versus imputed sample: total effect includes pathway via income change, direct effect blocks pathway via income

|  | **Complete cases** | | **Imputed sample (Table 1)** | |
| --- | --- | --- | --- | --- |
|  | **Total effect** (95% CI) | **Direct effect** (95% CI) | **Total effect** (95% CI) | **Direct effect** (95% CI) |
| **Odds ratio** | 1.72 (1.60, 1.84) | 1.58 (1.46, 1.70) | 1.66 (1.57, 1.76) | 1.55 (1.46, 1.66) |
| **% change** | 7.46% (6.40%, 8.52%) | 6.14% (4.98%, 7.30%) | 7.09% (6.21%, 7.97%) | 6.08% (5.13%, 7.03%) |
| **Unexposed prev.** | 17.53% (17.17%, 17.90%) | 17.96% (17.46%, 18.47%) | 18.24% (17.87%, 18.61%) | 18.58% (18.11%, 19.04%) |
| **PAF** | 17.71% | 14.29% | 16.49% | 13.90% |
| **% mediation** | 17.73% (9.12%, 26.34%) | | 14.22% (8.04%, 20.40%) | |
|  | n = 32,138; obs = 132,962 | | n = 45,497; obs = 202,297 | |

PAF = population attributable fraction. Complete cases are from observations with complete data on variables of interest from a wave and the wave preceding it.

### Table S7: The effect of two different measures of unemployment on likelihood of common mental disorder: total effect includes pathway via income change, direct effect blocks pathway via income

|  | **Reporting any paid work (primary analysis)** | | **ILO definition of unemployment (sensitivity analysis)** | |
| --- | --- | --- | --- | --- |
|  | **Total effect** (95% CI) | **Direct effect** (95% CI) | **Total effect** (95% CI) | **Direct effect** (95% CI) |
| **Odds ratio** | 1.66 (1.57, 1.76) | 1.55 (1.46, 1.66) | 2.60 (2.33, 2.90) | 2.37 (2.07, 2.71) |
| **% change** | 7.09% (6.21%, 7.97%) | 6.08% (5.13%, 7.03%) | 14.70% (12.62%, 16.77%) | 12.72% (10.34%, 15.10%) |
| **Unexposed prev.** | 18.24% (17.87%, 18.61%) | 18.58% (18.11%, 19.04%) | 16.47% (16.19%, 16.74%) | 16.99% (16.63%, 17.34%) |
| **PAF** | 16.49% | 13.90% | 31.27% | 27.53% |
| **% mediation** | **14.22% (8.04%, 20.40%)** | | **13.45% (3.37%, 23.53%)** | |
|  | n = 45,497; obs = 202,297 | | n = 26,971; obs = 106, 741 | |

ILO = International Labour Organisation. PAF = population attributable fraction. Sensitivity analysis is in complete cases and excludes participants whose labour force status did not meet the ILO definition of either ‘in employment’ or ‘unemployed’.

### Figure S1: Flowchart of sample selection and exclusion


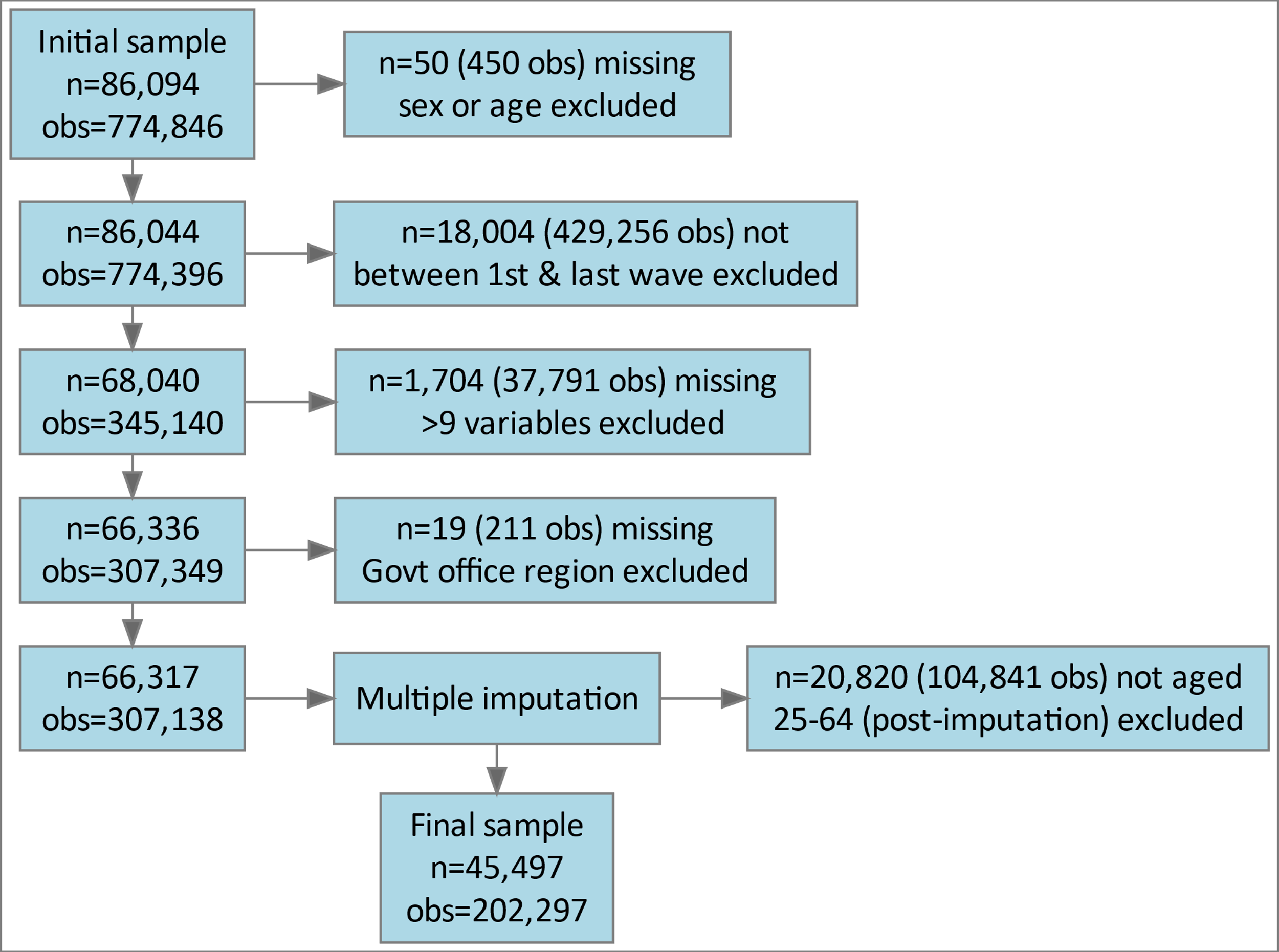

Supplement: Online supplement [file EMS182205-supplement-Online_supplement.docx]
